# A Hybrid Non-Ribosomal Peptide/Polyketide Synthetase Containing Fatty-Acyl Ligase (FAAL) Synthesizes the β-Amino Fatty Acid Lipopeptides Puwainaphycins in the Cyanobacterium *Cylindrospermum alatosporum*

**DOI:** 10.1371/journal.pone.0111904

**Published:** 2014-11-04

**Authors:** Jan Mareš, Jan Hájek, Petra Urajová, Jiří Kopecký, Pavel Hrouzek

**Affiliations:** 1 Institute of Microbiology AS CR, v.v.i., Department of Phototrophic Microorganisms – ALGATECH, Třeboň, Czech Republic; 2 Biology Centre of AS CR, v.v.i., Institute of Hydrobiology, České Budějovice, Czech Republic; 3 University of South Bohemia, Faculty of Science, Department of Botany, České Budějovice, Czech Republic; 4 University of South Bohemia, Faculty of Science, Department of Molecular Biology and Genetics, České Budějovice, Czech Republic; Indian Institute of science, India

## Abstract

A putative operon encoding the biosynthetic pathway for the cytotoxic cyanobacterial lipopeptides puwainphycins was identified in *Cylindrospermum alatosporum*. Bioinformatics analysis enabled sequential prediction of puwainaphycin biosynthesis; this process is initiated by the activation of a fatty acid residue via fatty acyl-AMP ligase and continued by a multidomain non-ribosomal peptide synthetase/polyketide synthetase. High-resolution mass spectrometry and nuclear magnetic resonance spectroscopy measurements proved the production of puwainaphycin F/G congeners differing in FA chain length formed by either 3-amino-2-hydroxy-4-methyl dodecanoic acid (4-methyl-Ahdoa) or 3-amino-2-hydroxy-4-methyl tetradecanoic acid (4-methyl-Ahtea). Because only one puwainaphycin operon was recovered in the genome, we suggest that the fatty acyl-AMP ligase and one of the amino acid adenylation domains (Asn/Gln) show extended substrate specificity. Our results provide the first insight into the biosynthesis of frequently occurring β-amino fatty acid lipopeptides in cyanobacteria, which may facilitate analytical assessment and development of monitoring tools for cytotoxic cyanobacterial lipopeptides.

## Introduction

Analogous to many other bacterial groups, cyanobacteria possess a unique biosynthetic apparatus capable of generating enormous structural diversity in various secondary metabolites [1]–[3]. Large multidomain enzymes of non-ribosomal peptide synthetase (NRPS) machinery and polyketide synthetases (PKS) can be reassembled to generate an almost infinite number of chemical structures. Some of the end-products of this machinery have been found to be important pharmaceuticals; by contrast, others are recognized as important environmental toxins. In cyanobacteria, hepatotoxic microcystins [4], nodularins [5], cylindrospermopsin [6] and neurotoxic alkaloids anatoxin [7], and saxitoxins [8] are produced by the NRPS/PKS pathways. This finding has initiated intensive research on secondary metabolite biosynthesis in cyanobacteria. Moreover, these results also brought about the possibility of detecting cyanotoxins using molecular markers [6]–[9].

As one of the less explored cyanobacterial secondary metabolites, cyclic lipopeptides may be important due to their broad biological effects in various organisms, which also raises questions on their possible toxicity to humans [10], [11]. Thus far, approximately 80 structural variants derived from several core structures have been isolated from cyanobacteria. The peptide cycle may comprise four amino acid residues, as in the case of anabaenolysins [12], or up to 14 amino acid residues, as in malevamides [13]. The lipidic part of the molecule may be formed by a modified fatty acid (FA) and connected to the ring by a carboxyl group via peptide bond formation, as in hassallidins [14]. Alternatively a β-amino FA may be incorporated into the cycle by two peptide bonds formed from a carboxyl group and a β-amino group, as found in the majority of described cyanobacterial lipopeptides. Some 16 core structures of cyanobacterial β-amino FA lipopeptides have been identified thus far. They display a broad range of bioactivities including cytotoxicity, as in the case of laxaphycins [15], hormothamnin A [16], minutissamides [17], pahayokolides [18] and lyngbyacyclamides [19], or antifungal and/or antibacterial activity [20]–[25].

The chemical structures of cyanobacterial lipopeptides share some similarities with those produced by other bacterial groups. The well-known bacterial lipopeptide surfactin and members of the iturin lipopeptide family (bacillomycin, mycosubtilin, lichenysin) comprise a seven-member ring of D- and L-amino acids and a FA side chain of varying length [26]–[30]. In the surfactin molecule, the FA chain is connected to the ring by a peptide bond on the carboxyl side and an ester bond between the β-hydroxy group of the unusual FA and the isoleucine of the peptide cycle. In the case of iturin family members, β-amino FA is present; thus, it is incorporated via a standard peptide bond, similar to the majority of cyanobacterial lipopeptides. Finally, the FA chain may be connected via an ester bond to the linear part of the peptide molecule, as in fengycin [31], which is the structural analog of the cyanobacterial metabolite hassallidin. The majority of reported bacterial lipopeptides have been isolated from *Bacillus subtillis* and display a broad range of biological activities including antibiotic and antifungal effects [32].

The biosynthesis pathway of small bacterial lipopeptides was initially elucidated for mycosubtilin, an iturin lipopeptide family member produced by *Bacillus subtillis* ATCC6633 [33]. Insertional mutagenesis of an operon encoding four ORFs (*fenF*, *mycA-C*) proved the operon to be responsible for mycosubtilin biosynthesis. The operon encodes MycA, a hybrid enzyme combining domains involved in polyketide, FA and peptide synthesis. This multifunctional enzyme activates a FA residue, elongates it, modifies it by placing an amino moiety on the β position, catalyzes asparagine addition and passes it on for further synthesis of the peptide cycle by two peptide multisynthetases (MycB and MycC). The mycosubtilin biosynthesis is finalized by forming a peptide bond between the β-amino group of the FA and the last asparagine of the cycle. Similarly, in the case of surfactin [34], biosynthesis is initiated by transfer of a β-hydroxy FA mediated by acyl transferase SrfD. Eventually, the peptide cycle is closed by forming an ester bond of the hydroxyl with a leucine of the peptide backbone [35].

In cyanobacteria, an operon encoding the enzymes involved in the synthesis of the hassallidin type of lipopeptides has been recently characterized [36]. The authors clarified the formation of the peptide backbone and predicted the modules responsible for glycosylation and acetylation of the molecule. As mentioned above, apart from the hassallidin (sfingomycin) type of lipopeptides, cyanobacteria produce structurally diverse cyclic peptides containing β-amino fatty acids that possess cytotoxic and antifungal bioactivities and that may potentially be toxic to humans. Thus, knowledge of their biosynthetic pathways is essential for the understanding of chemical variability in their structures and for providing screening markers for toxic lipopeptides in cyanobacteria.

In this report, we describe the characterization of the biosynthetic gene cluster encoding the cytotoxic cyanobacterial lipopeptides puwainaphycins and describe novel structural variants of puwainaphycins synthesized by a single enzyme complex. For the first time, we identify an operon of a β-amino lipopetide in cyanobacteria, comparing its features to the lipopetide variants produced, and discussing the presence of genes that may be essential for the biosynthesis of cyclic bacterial lipopetides in general.

## Materials and Methods

### Cultivation of cyanobacterial biomass

The uni-cyanobacterial strain *Cylidrospermum alatosporum* CCALA 988 (C24/1989), previously shown to be a typical member of the *Cylidrospermum* cluster [37], was cultivated in 350-mL glass tubes in liquid Allen Arnon medium [38] and bubbled with 2% CO_2_-enriched air at a constant temperature of 28°C, under 50 W.m^−2^ continuous illumination. Following 5–7 days of cultivation, the culture was harvested by centrifugation, stored at –70°C and freeze-dried.

### Extractions

Eight grams of lyophylized *Cylindrospermum alatosporum* biomass was extracted using methanol/water (70/30, v/v) for 1 hour and then centrifuged at 1920 *g*. Supernatant was collected, and the volume was reduced using a rotary evaporator where the temperature did not exceed 38°C. The final solution was extracted in a glass funnel using H_2_O:n-hexan (4:1). The water phase was collected and concentrated under the same conditions using a rotary evaporator and finally dissolved in 2 mL pure MeOH for high-performance liquid chromatography (HPLC) purification.

To quantify 4-methyl-Ahdoa-PUW-F and 4-methyl-Ahtea-PUW-F (Puwainaphycin F), 200 mg of freeze-dried *Cylindrospermum alatosporum* biomass was extracted using methanol/water (70/30, v/v) solution for one hour. Following centrifugation at 1920 *g* for 10 minutes, the supernatant was concentrated under a vacuum at 38°C to dryness, and the residue was then dissolved in 1 mL of methanol/water (70/30, v/v) solution. After centrifugation, the pellet was extracted once again using the same procedure. The solutions were 10x diluted prior to LCMS analysis. All samples were prepared in triplicate. Purified compounds used as standards were prepared in our laboratory.

### HPLC purification

The concentrated extract was purified using a HPLC-MS Agilent 1260 Infinity series equipped with preparative pumps, a multiwavelength detector, automatic fraction collector and a mass spectrometer ESI-Quadrupole (Agilent 6120). The first step was performed using a semi-preparative Eclipse XDB-C18 column (9.4×250 mm) with methanol/water gradient, see Table S1, and at a flow rate of 3.5 mL.min^−1^. Fractions were collected using an automatic fraction collector at 1-min intervals. The fractions containing puwainaphycin analogs with *m/z* 1118.6, 1134.6, 1146.6, 1152.6, 1162.6 and 1180.6 were collected in separate vials. A second purification step was performed using a semi-preparative Reprosil 100 Phenyl column (250×8 mm) with methanol/water gradient, see Table S2, at a flow rate of 2.5 mL.min^−1^. Desired fractions were collected again using the automatic fraction collector. The fractions obtained were dried under nitrogen.

### HPLC–high-resolution mass spectrometry (HRMS) analysis and MS/MS experiments

*Cylindrospermum alatosporum* 70% methanolic extracts were analyzed using a Thermo Scientific Dionex UltiMate 3000 UHPLC+ equipped with a diode-array detector. Separation of compounds was performed on a reversed-phase Phenomenex Kinetex C18 column (150×4.6 mm, 2.6 µm) using H_2_O (A)/acetonitrile (B), both of which contained 0.1% HCOOH as a mobile phase, at a flow rate of 0.5 mL.min^−1^. The gradient was as follows: A/B 85/15 (0 min), 85/15 (in 1 min), 0/100 (in 20 min), 0/100 (in 25 min) and 85/15 (in 30 min). The HPLC was connected to a Bruker Impact HD high-resolution mass spectrometer with electrospray ionization. The following settings were used: dry temperature, 200°C; drying gas flow, 12 L.min^−1^; nebulizer, 3 bar; capillary voltage, 4500 V; endplate offset, 500 V. The spectra were collected in the range 20–2000 *m/z* with a spectra rate of 3 Hz. Spectra were calibrated using both LockMass 622 internal calibration solution and sodium formate clusters at the beginning of each analysis. The summary formulas of molecular peaks obtained were calculated using Smart Formula in Bruker Compass DataAnalysis software (version 4.2). The fractions obtained from preparative chromatography were used for manual direct infusion to a Bruker Impact HD spectrometer at the following settings: dry temperature, 180°C; drying gas flow, 4 L.min^−1^; nebulizer, 0.4 bar; capillary voltage, 4500 V; endplate offset, 500 V. The spectra were collected in the range 20–2000 *m/z* at a spectra rate of 3 Hz. The fractions were dissolved in methanol containing 0.05% formic acid for more effective ionization, and molecular peaks were isolated in quadrupole with window 3 *m/z*, and the collision energy was set manually from 0 to 150 eV.

### Nuclear magnetic resonance

Pure samples were diluted in 500 µL of DMSO. For NMR measurements, a 700 MHz AvanceIII spectrometer with an Ascend magnet and a TCI cryoprobe for high sensitivity and high resolution was used. For structure determination, ^1^H, ^1^H^13^C heteronuclear single quantum coherence NMR spectroscopy (HSQC), ^1^H^15^N HSQC and TOSCY spectra were recorded. Data were analyzed using Bruker TopSpin 3.0 and MestReNova 6.0.

### Molecular and bioinformatic analysis

Single filaments of *Cylindrospermum alatosporum* CCALA 988 were isolated using the glass capillary technique [39] using an Olympus CX31 microscope (×200–400 magnification) to exclude minor bacterial contaminants from the subsequent molecular analyses. Suitable filaments were washed using 10 droplets of TE buffer, and finally, each was placed in a single 0.2-mL PCR tube. All instruments and the TE buffer were sterilized by autoclaving and UV, and the microscope work area was cleaned using 100% ethanol and treated with UV light for one hour prior to the start of the isolation. After freezing (–20°C) for 3 days and thawing for the initiation of cell lysis, the *C. alatosporum* filaments were used as a template for whole-genome amplification (WGA). Multiple displacement amplification (MDA) with Phi29 polymerase was conducted using Repli-g Mini Kit (Qiagen) according to the manufacturer’s protocol. MDA products were tested by PCR for the cyanobacterial 16S rRNA gene [40]. Positive samples (16 MDA products) were then pooled to create a template for WGA sequencing, to reduce possible unequal MDA amplification throughout the genome. The DNA was sent for commercial *de* *novo* genome sequencing (Macrogen, Inc.) using Illumina HiSeq2000 (Illumina) with a ∼500-bp insert Pair-End library and 100-bp reads (approximately 9 Gbp data yield). The sequence data were deposited in the NIH Sequence Read Archive (http://trace.ncbi.nlm.nih.gov/Traces/sra/) and they are accessible under the NCBI Bio Project PRJNA261005, BioSample SRS700947.

Raw data from *de* *novo* WGS were assembled using CLC Bio Genomics Workbench (CLC Bio). The genomic contigs were loaded into Geneious Pro R6 (Biomatters; available from http://www.geneious.com) and investigated for NRPS genes using blastp searches with several known cyanobacterial amino acid adenylation domains (A-domains) as queries. Contigs yielding high similarity hits for A-domains were then analyzed using the Glimmer 3 [41] algorithm to discover putative ORFs. Functional annotation of the ORFs (Table S3) was conducted by applying a combination of blastp searches against the NCBI database, and by HMMER 3 [42] and InterPro Scan 5 [43] searches in available protein databases. The organization of the individual NRPS/PKS modules and A-domain amino-acid specificity was assessed using PKS/NRPS Analysis [44], NRPSpredictor2 [45] and NaPDoS [46] online applications.

The predicted biosynthetic pathway for puwainaphycins contained two minor gaps in the DNA sequence, which were covered by Sanger sequencing (SeqMe, Dobříš, Czech Republic) using all combinations of custom specific primers located near to contig ends. The complete sequence of the predicted biosynthetic pathway was uploaded to the NCBI GenBank database under accession number KM078884.

## Results

### Predicted biosynthetic pathway

**Figure 1:**
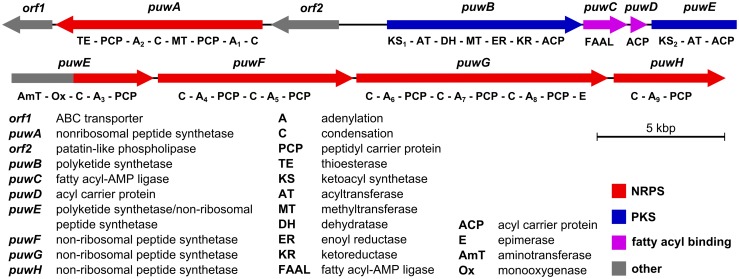
Gene arrangement, functional annotation and domain structure of the *puw* gene cluster (56.7 kbp) from *Cylindrospermum alatosporum* CCALA 988.

After the final assembly of Illumina and Sanger sequencing data, the predicted puwainaphycin biosynthesis pathway was recovered in the middle of a ∼100-kbp long contig, with >20 kbp flanks at each side to ensure that the full pathway was sequenced. The putative biosynthetic gene cluster was 56,728 bp long and comprised 10 protein-coding ORFs. The ORFs were transcribed starting from a bi-directional promoter region, with *orf2*, *puwA* and *orf1* transcribed in one direction and *puwB*-*H* in the opposite direction (Figure 1). The functional annotation of the individual enzymes and catalytic domains is summarized in Figure 1 and Table S3.

**Figure 2:**
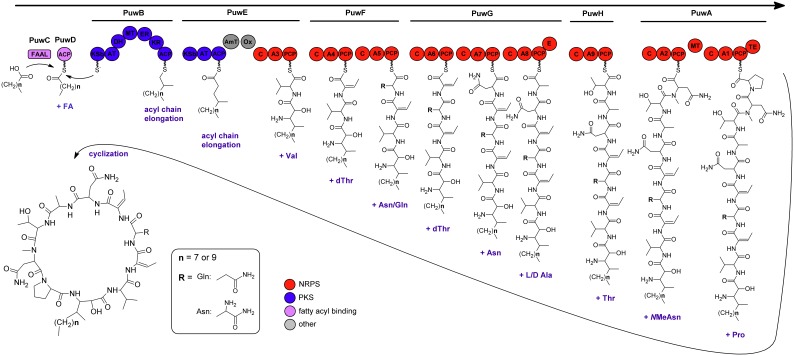
Proposed biosynthesis of puwainaphycins. A, adenylation domain; ACP, acyl carrier protein; AmT, aminotransferase; AT, acyltransferase; C, condensation domain; DH, dehydratase; E, epimerase; ER, enoylreductase; FAAL, fatty acyl-AMP ligase; MT, methyltransferase; NRPS, non-ribosomal peptide synthetase; KR, ketoreductase; KS, ketosynthetase; Ox, monooxygenase; PCP, peptidyl carrier protein; PKS, polyketide synthetase; TE, thioesterase.

The reconstructed course of puwainaphycin biosynthesis, as visualized in Figure 2, starts with the *puwC* gene product. This protein contains conserved catalytic domains typical for fatty acyl-AMP ligases (FAALs), enzymes that activate a fatty acyl by adenylation to subsequently serve as a substrate for polyketide synthetases. PuwC also showed a relatively high amino acid sequence identity (approximately 60%) to several annotated cyanobacterial fatty-acyl acyl carrier protein (ACP) ligases. After activation of the fatty acyl and its ligation to the first ACP encoded by *puwD*, it is forwarded directly to the PKS machinery. The first unimodular PKS (encoded by *puwB*) contains a canonical sequence of acyltransferase, dehydratase, methyltransferase, enoylreductase and ketoreductase domains, resulting in an α-methylated product. The next enzyme (PuwE) is an unusual hybrid comprising one PKS module, a putative aminotransferase and a monooxygenase domain, and a terminal single NRPS module. The PKS module solely elongates the acyl chain with no additional modifications; however, the aminotransferase and monooxygenase located between the PKS and NRPS modules of the enzyme apparently catalyze the tailoring of the chain into a 3-amino-2-hydroxy-4-methyl-acyl typical for this group of lipopeptides. The predicted aminotransferase and monooxygenase domains show sequence similarity (50–70%) to several class-III aminotransferases and flavin-utilizing monooxygenases from cyanobacteria. The terminal NRPS module includes an A-domain activating valine as the first amino acid member of the oligopeptide ring. Valine was chosen as the most probable amino acid based on a nearest-neighbor search in NRPSPredictor2 (90%); the closest blast hits were uncharacterized cyanobacterial A-domains. Subsequently, the intermediate is transferred to another NRPS enzyme (PuwF) that comprises two modules, each adding one amino acid - dehydrothreonine and asparagine/glutamine. The A-domain putatively responsible for the incorporation of dehydrothreonine shows an amino acid-specific structural motif with considerable similarity to known threonine-activating A-domains (100% threonine in nearest-neighbor search, 64–66% pairwise identity to McnB and OciE, which are involved in cyanopeptolin synthesis in *Microcystis* and *Planktothrix*). Because no additional tailoring enzymes with amino-acid dehydration function were found in the pathway, this domain is possibly specific directly for dehydrothreonine. The second A-domain of PuwF was recovered as specific (100%) to asparagine in the nearest-neighbor analysis; however, the closest, weakly similar blast hit (54% amino acid sequence identity) was the glutamine-activating McnA protein included in cyanopeptolin biosynthetic pathways. Considering the puwainaphycin F/G variants reported in this study, the A-domain appears to accept both asparagine and glutamine as a substrate at a defined rate, as further demonstrated by MS measurements. The following biosynthesis steps are catalyzed by the *puwG* product that comprises three NRPS modules predicted to incorporate dehydrothreonine, asparagine and alanine. The dehydrothreonine-incorporating module is highly identical (97%) to that in PuwF, as described earlier. The other two modules activate asparagine and alanine, based on a nearest neighbor search (100%). The first shows similarity (55%) to the asparagine-activating A-domain in NosC nostopeptolide synthetase from *Nostoc*, and the second is similar (63% identity) to the alanine-activating domain in the jamaicamide synthesis gene JamO (*Lyngbya majuscula*). The last module of PuwG includes an epimerization domain consistent with the occurrence of both optical isomers (L/D-alanine) in the puwainaphycin molecules. The addition of threonine, the next member of the oligopeptide ring, is catalyzed by another protein (PuwH) that possesses a single NRPS module (100% threonine in a nearest-neighbor search, 65% identity with threonine-incorporating OciE). The last enzyme involved in the peptidyl elongation is PuwA. It clearly comprises two NRPS modules, the first containing a specific motif in the A-domain predicted to activate asparagine (90% asparagine in a nearest-neighbor search, 57% identity with NosC asparagine-activating A-domain) and a methyltransferase domain obviously linked to N-methylation of that asparagine. The second module is predicted to incorporate proline (80% proline in a nearest-neighbor search, 63% identity with NpnC proline-activating domain involved in nostophycin synthesis) and a thioesterase domain in its terminal part that cleaves the finished puwainaphycin chain from the peptidyl carrier protein, thus promoting its cyclization.

The function of the *orf2* product could not be confidently linked to any step in puwainaphycin biosynthesis. Its amino acid sequence shows considerable similarity (40–55%) to cyanobacterial patatin-like family phospholipases, enzymes capable of fatty-acyl cleavage from membrane phospholipids. The last ORF that appears to be part of the biosynthetic cluster is *orf1*. The encoded protein shows similarity (53–58%) to the ABC-transporter family proteins in cyanobacteria; thus, it may be involved in the transport of puwainaphycin to its destination in the cell. No specific enzymes linkable to chlorination and hydroxylation of the fatty acyl chain were noted in the puwainaphycin biosynthetic cluster or its vicinity, and no protein coding sequence with predicted halogenase activity was recovered from the WGS data. Thus, the mechanism of these modifications for a minor part of the puwainaphycin produced remains unexplained by the genomic data.

### High-resolution HPLC-MS analysis of puwainaphycins analogs

**Figure 3:**
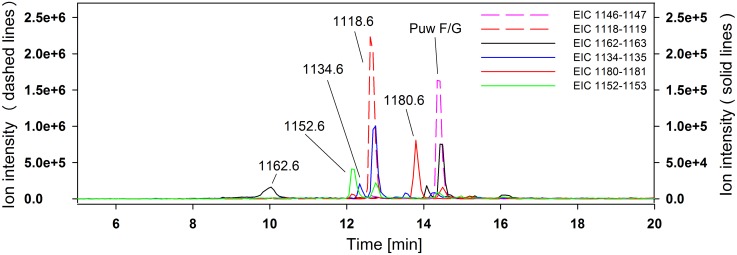
HPLC-HRMS analysis of *Cylindrospermum alatosporum* CCALA 988 extract. Extracted ion chromatograms of puwainaphycin analogs are shown, indicating their *m/z* values using different scales for major (left axis) and minor variants (right axis).

**Figure 4:**
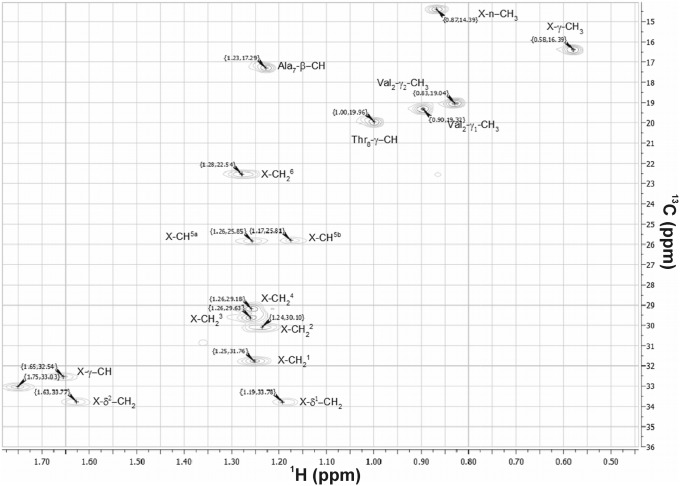
^1^H^13^C HSQC spectrum of 4-methyl-Ahdoa puwainaphycin F at a range of shifts 15–34 ppm for C^13^ and 0.5–1.5 ppm for ^1^H. Assignment of the peaks shows an aliphatic chain containing 7 carbons (X-CH_2_^1–6^, X-CH_3_) and additional peaks X-CH_2_^7–8^ found in 4-methyl-Ahtea puwainaphycin F are missing.

**Figure 5:**
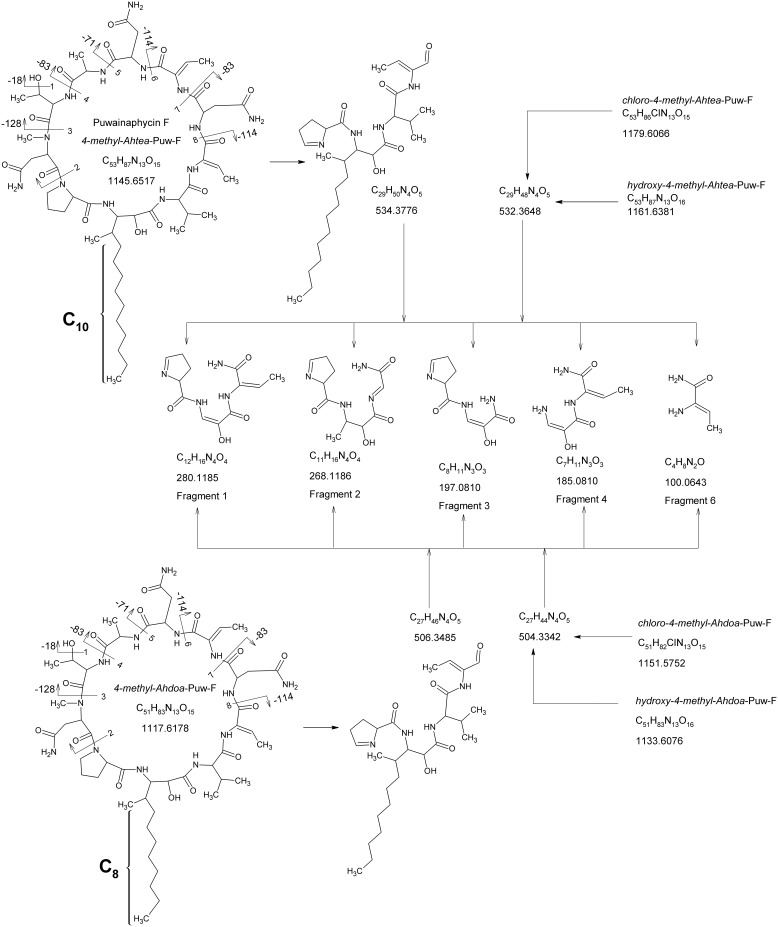
Fragmentation of 4-methyl-Ahdoa and 4-methyl-Ahtea puwainaphycin F and their chlorinated and hydroxylated analogs. The observed fragmentation losses are denoted by the corresponding amino acid residues. The diagnostic fragments common to all puwainaphycin analogs are marked as Fragments 1–6.

The HPLC-HRMS analysis of crude *Cylindrospermum alatosporum* extract revealed about 30 pseudomolecular ions in the region 1050–1200 Da, which were attributed to putative puwainaphycin analogs. Puwainaphycin F (*4-methyl-Ahtea*-PUW-F), together with the congener of *m/z* 1118.6, were found to be dominant based on UV and MS detection, other variants were observed in trace amounts based on their MS signals (Figure 3B). Of these we have selected eight more prominent peaks for purpose of this study. Their exact masses were measured with high precision (0.0–4.9 ppm), enabling calculation of the elemental composition (Table 1). The MS/MS experiments provided product ions corresponding to identical losses of *N*-methyl asparagine (Δ 128), dehydrated threonine (Δ 83), alanine (Δ 71), asparagine (Δ 114) and dehydrothereonine (Δ 83) in all compounds detected (Figure S1). Furthermore, the fragmentation pattern of the pseudomolecular ions with *m/z* 1118.6, 1152.6, 1180.6, 1134.6 and 1162.6 showed clear loss of an additional asparagine and thus have the same amino acid composition as *4-methyl-Ahtea-*PUW-F (Figure S1A, B). The loss of glutamine was recognized in compounds *m/z* 1126.6, 1132.6, 1176.6, 1194.6, and 1166.6; thus, their amino acid sequence is identical to puwainaphycin G (*4-methyl-Ahtea-*PUW-G; Figure S1C, D). Moreover, the fragmentation of all of these compounds led to the formation of the diagnostic fragments *m/z* 101.0, 186.1, 198.1, 269.2, 281.2, which were identical for all precursor ions and further confirm the identical primary structure of the cyclic part of the molecule. The ion 281.2 corresponded to fragment C_12_H_16_N_4_O_4_+H^+^ (Pro-X^1^-Val-dThr, where X^1^ corresponds to the 3-amino-2-oxopropanoyl fragment of the 3-amino-2-hydroxy-4-methyl-fatty acid but where the major part of the aliphatic chain was missing. The remaining diagnostic fragments were derived from ion 281.2. These results confirm that non-identical parts of the puwainaphycin variants are situated in the unusual FA aliphatic chain whereas the remaining part of the molecule is identical for all the puwainaphycin analogs, based on the PUW-F/PUW-G structures. Indeed, within the fragmentation spectra of *4-methyl-Ahtea*-PUW-F and its analog 1118, the intense fragment ions *m/z* 535 and 507, corresponding to sequence Pro-X-Val-DhB (with X as modified FA), were recognized (Figure 4). The mass difference of 28.0291 Da corresponded well with –(CH_2_)_2_ on the FA chain (2.2 ppm), and this mass difference was noted in all fragments containing the FA residue (Table S4 A–F). The presence of 3-amino-2-hydroxy-4-methyldodecanoic (*4-methyl-Ahdoa*) instead of 3-amino-2-hydroxy-4-methyltetradecanoic acid (*4-methyl-Ahtea*) in the congener 1118.6 was further confirmed by NMR measurements (see Table S5). The 2D NMR spectra (Figure S2) of congener 1118.6 were almost identical to the previously measured NMR spectra of *4-methyl-Ahtea*-PUW-F [10]. The only non-identical part of the spectrum was noted in the HSQC region set by a ^13^C shift of 15–34 ppm and a ^1^H shift of 0.5–1.5 ppm. Although in *4-methyl-Ahtea*-PUW-F ^1^H^13^C, the HSQC crosspeaks X-CH_2_^1^ to X-CH_2_^9^ were identified, in the case of *4-methyl-Ahdoa*-PUW-F, the crosspeaks X-CH_2_^8^ and X-CH_2_^9^ were clearly missing (see Figure 5). An identical MS/MS fragmentation pattern was also provided by the ion 1132.6 (Figure S1 C, D), a structural analog of *4-methyl-Ahtea*-PUW-G; thus, the *4-methyl-Ahdoa*-PUW-G was confirmed as the variant of puwainaphycin G with tetradecanoic acid replaced by dodecanoic acid (see Table S4). The ^1^H^13^C HSQC crosspeaks unique for Gln^4^ in *4-methyl-Ahdoa*-PUW-G were also identified in the sample; however, the full assignment of this variant was not successful due to overlapping of very similar signals with *4-methyl-Ahdoa*-PUW-F and its low concentration (Table S5). Additionally, in some of less intensive molecular ions observed, the fragmentation analysis suggests presence of analogs with shorter (<C12) and longer (>C14) FA chains (data not shown).

**Table 1:** Exact masses of puwainaphycin analogs and their elemental composition.

m/z	Molecular Formula	Puwainaphycin Variants
1118.6248	C_51_H_83_N_13_O_15_+H^+^	*4-methyl-Ahdoa*-Puw-F
1132.6355	C_52_H_85_N_13_O_15_+H^+^	*4-methyl-Ahdoa*-Puw-G
1134.6146	C_51_H_83_N_13_O_16_+H^+^	*hydroxy-4-methyl-Ahdoa*-Puw-F
1148.6342	C_52_H_85_N_13_O_16_+H^+^	*hydroxy-4-methyl-Ahdoa*-Puw-G
1146.6517	C_53_H_87_N_13_O_15_+H^+^	*4-methyl-Ahtea*-Puw-F **(Puwainaphycin F)**
1160.6727	C_54_H_89_N_13_O_15_+H^+^	*4-methyl-Ahtea*-Puw-G **(Puwainaphycin G)**
1152.5822	C_51_H_82_ClN_13_O_15_+H^+^	*chloro-4-methyl-Ahdoa*-Puw-F
1166.5953	C_52_H_84_ClN_13_O_15_+H^+^	*chloro-4-methyl-Ahdoa*-Puw-G
1162.6451	C_53_H_87_N_13_O_16_+H^+^	*hydroxy-4-methyl-Ahtea*-Puw-F
1176.6648	C_54_H_89_N_13_O_16_+H^+^	*hydroxy-4-methyl-Ahtea*-Puw-G
1180.6136	C_53_H_86_ClN_13_O_15_+H^+^	*chloro-4-methyl-Ahtea*-Puw-F
1194.6296	C_54_H_88_ClN_13_O_15_+H^+^	*chloro-4-methyl-Ahtea*-Puw-G

MS/MS spectra were also obtained from other puwainaphycin congeners that showed a constant shift in mass of the fragments possessing FA moiety compared with other congeners. In the pseudomolecular ions 1180.6 and 1152.6, the fragments containing FA chains differed by 33.96 Da from *4-methyl-Ahtea-*PUW-F and 4*-methyl-Ahdoa-*PUW-F, respectively. This mass difference fits with replacing one hydrogen bonded on the FA chain by chlorine (2.2 ppm). The presence of chlorine on the aliphatic FA chain has already been described in puwainaphycin variants C–D [45]. The fragmentation of ion 1180.6 showed not only a constant shift in mass but also a new fragment ion of *m/z* 533.4. This fragment ion may be assigned to Pro-X^1^-Val-DhB, where X^1^ represents monounsaturated 4-methyl-Ahtea formed by the loss of –Cl and subsequent double bond formation. Analogously, the ion of *m/z* 505.3 observed in the fragmentation spectrum of ion 1152.6 corresponds well with Pro-X^2^-Val-DhB, where X^2^ is monounsaturated 4-methyl-Ahdoa (Figure 4, Table S3 C, D). Identical fragmentation leading to similar product ions was observed in PUW-G analogs (1194.6 and 1166.6) and thus also chlorinated *4-methyl-Ahdoa*-PUW-G and *4-methyl-Ahtea*-PUW-G were confirmed to be produced by the *Cylindrospermum* strain. Finally, the presence of hydroxylated variants of all puwainaphycin congeners may be explained by the constant shift of 16 Da on the lipidic chain and the formation of a fragment with monounsaturated 4-methyl-Ahdoa/Ahtea of *m/z* 533.4 and 505.3 (Figure 4, Table S3 E, F).

### Quantitative analysis and ratio of puwainaphycin variants

Quantitative analysis was performed using a calibration curve with a range of concentrations of *4-methyl-Ahtea-*PUW-F and *4-methyl-Ahdoa-*PUW-F of 10–500 µg.mL^−1^. The MS peaks, belonging to *4-methyl-Ahdoa-*PUW-F and *4-methyl-Ahtea-*PUW-F, were eluted at 12.9 and 14.6 min, respectively. The content of *4-methyl-Ahdoa-*PUW-F and *4-methyl-Ahtea-*PUW-F in freshly harvested biomass was 13.9±7.3 mg.g^−1^ of dry biomass and 7.7±3.9 mg.g^−1^ of dry biomass, respectively. The content of the two congeners was greater compared with biomass stored seven years at laboratory temperature, the concentrations of which were 9.8±4.0 mg.g^−1^ of dry biomass and 5.7±2.1 mg.g^−1^ of dry biomass. The ratio in content of both congeners remained almost constant during storage. Although the ratio of *4-methyl-Ahdoa-*PUW-F/*4-methyl-Ahtea-*PUW-F was 1.83 in freshly harvested biomass, the ratio changed to 1.74 in the aged biomass. Furthermore, the ratio of asparagine vs. glutamine variants of the different congeners was verified by HPLC-HRMS. The ratio was calculated by comparing the integrated area of peaks on an extracted ion chromatogram ([M+H]^+^, [M+Na]^+^, [M+2H]^2+^) of every single congener. The proportion of asparagine vs. glutamine variants was similar for all puwainaphycin analogs and varied from 1:0.15 to 1:0.23 (Table 2). However, the lower ratio was observed in the variants that were low in concentration; thus, the peak integration may have contained greater error.

**Table 2:** Relative ratio of asparagine^4^- vs. glutamine^4^-containing puwainaphycin variants.

Fatty acid	EIC PUW-F[milions of ions]	EIC PUW-G[milions of ions]	Ratio
*4-methyl-Ahtea*	38.97	8.3	1:0.21
*4-methyl-Ahdoa*	48.94	11.28	1:0.23
*chloro-4-methyl-Ahtea*	8.2	1.73	1:0.21
*chloro-4-methyl-Ahdoa*	6.77	1.35	1:0.20
*hydroxy-4-methyl-Ahtea*	0.84	0.17	1:0.20
*hydroxy-4-methyl-Ahdoa*	1.81	0.28	1:0.15

The only deviation from the ratio 1:0.2 was recorded in the case of hydroxy-4-methyl-Ahdoa PUW F/G, which may be explained by its low ion intensity and the presence of coeluting compounds.

## Discussion

Utilizing a combination of DNA sequencing, bioinformatics analyses and analytical chemistry (HRMS, NMR) approaches, we elucidated the genetic background and probable course of biosynthesis of the cytotoxic cyanobacterial lipopeptides puwainaphycins [10]. Puwainaphycins represent a large group of cyanobacterial β-amino FA containing lipopeptides with unknown biosynthetic pathways [12], [13], [15], [16], [18], [22], [23], [25], [47]. Thus, our results are the first step in clarifying the origin of an entire family of natural secondary metabolites. Some of these compounds have strong membrane disruption effects on human cells [10]–[12]. Interestingly, puwainaphycins possess some structural traits analogous to antibiotics of the iturin family produced by *Bacillus subtilis*, the biosynthesis of which has already been characterized [33], [48], [49]. However, the β-amino FA linked by two peptide bonds, which is present in the iturin family of antibiotics, lacks some of the modifications typical for puwainaphycins and the majority of cyanobacterial lipopeptides. This especially relates to the 2-hydroxy and 4-methyl functional groups. The puwainaphycin biosynthetic operon also shows features similar to that of the iturin family members iturin A, mycosubtilin and bacillomycin D. Most importantly, the AMP-dependent fatty acyl ligase (FAAL) and aminotransferase domains present in MycA, ItuA and BamA have been proposed to be responsible for the formation of the β-amino FA part of the molecule [33], [48], [49]. The enzyme was reported to also include a PKS and NRPS module in a single ORF in addition to FAAL and aminotransferase. In the puwainaphycin cluster, the organization is highly divergent from that of the iturin family. The FAAL enzyme and the ACP, to which the FA is ligated at the very beginning of puwainaphycin synthesis, are encoded in separate ORFs (*puwC*-*D*), whereas the PKS subunit (PuwB) that elongates and methylates the fatty acyl is another gene. Moreover, the next ORF in the biosynthetic cascade (*puwE*) encodes a hybrid PKS/NRPS enzyme that comprises the aminotransferase and oxygenase domains that modify the methylated fatty acyl into a 3-amino-2-hydroxy-4-methyl-acyl typical for this family of lipopeptides. Based on HRMS measurements, a small portion of the puwainaphycin variants produced was found to be chlorinated or hydroxylated on the fatty acyl chain. This type of halogenation has been repeatedly reported from peptides possessing FA chain in cyanobacteria [1], [17], [50] including puwainaphycins [47]. In unusual chlorinated lipopetides barbamides, the chlorination was associated with a halogenase encoded within the biosynthetic pathway in *Lyngbya majuscula* [51]. However, as demonstrated for a linear lipopeptide oscillaginin A in *Planktothrix rubescens*, chlorination of the FA chain may also occur when no known type of halogenase is noted in either the biosynthetic cluster or the entire genome [52]. Similarly, in the case of the puwainaphycins, the mechanism of halogenation in these compounds remains unexplained. Perhaps a non-specific post-synthesis mechanism is involved, as further supported by the relatively low ratio of chlorinated puwainaphycin forms. However, the hydroxylation may be performed by a number of oxygenases putatively expressed by *Cylindrospermum*. Nevertheless, none were predicted as a standalone dedicated enzyme or catalytic domain within the *puw* cluster.

Based on the evidence presented above, we can conclude that all 12 detected puwainaphycin congeners produced by *Cylindrospermum alatosporum* CCALA 988 are synthesized by a single NRPS/PKS synthetase. The chemical structure of the previously characterized puwainaphycins A-E is similar to the F and G variants studied hereby, maintaining a 10-membered ring and sharing valine, dehydrothreonine, N-methylasparagine, and proline in the positions 2, 3, 9 and 10, respectively. However in the positions 4–8 the aminoacid residues can vary. In the position 4 asparagine/glutamine is replaced by threonine in puwainaphycin A-E. The position 5, which is occupied by dehydrothreonine in puwainaphycin F and G, is held by threonine or valine in puwainaphycin A-E. Alanine residue (position 6) in puwainaphycin F and G is replaced by glycine, and finally the threonine residue in the position 7 is replaced by O-methyl threonine in puwainaphycin A-E. Although this is the first report of a β-amino FA lipopeptide operon in cyanobacteria, we suggest that this diversity is most likely obtained by the replacement of the individual NRPS modules within the synthetase with modules activating alternative amino acids. The variation between asparagine and glutamine in the third amino acid position, the only difference between puwainaphycin F and G core cyclic structures, is most likely caused by extended substrate specificity of the amino acid activation domain of the PuwF subunit of the NRPS. Due to high 3D structural similarity, glutamine and asparagine may be complementary to the active site of the enzyme and therefore the extended substrate specificity is not surprising. The multispecificity of several adenylation domains has been shown already [50], [53]. The HPLC-MS data further showed that all variants based on the core cyclic structure of puwainaphycin F and G are produced at a ratio of 1:0.150.23. This ratio is consistent with previously published data obtained by NMR on mixed fractions of the F and G variant where the molar ratio was noted to be 1:0.24 [10]. Thus, we propose that the adenylation domain of PuwF incorporates one glutamine per four asparagine molecules. A similar mechanism most likely plays a role in the addition of the FA. Apparently, enanthic (C7) or pelargonic (C9) acid may be activated by the FAAL domain and subsequently elongated and modified to the final 3-amino-2-hydroxy-4-methyl-fatty acid. Based on quantitative HPLC-HRMS analyses, the *4-methyl-Ahdoa*-PUW-F and *4-methyl-Ahtea*-PUW-F are produced in a ratio of approximately 1:0.5, which reveals the different rate of enanthic and pelargonic acid incorporation. Multispecificity of the FAAL domain may also be clearly observed in the biosynthesis of iturin family members, where congeners differing in FA length were reported. The reported length of the FA ranges are C14C16 for iturin and bacillomycin and C16–C17 for mycosubtilin [32].

Because this is the first report of a complete biosynthetic pathway for lipopeptides containing β-amino FA in cyanobacteria, a direct comparison of biosynthetic steps employed in the synthesis of other lipopeptide variants in cyanobacterial cells is impossible. In the recently suggested biosynthesis of hassallidins, little explanation was provided on the mechanism of incorporation of the FA into the peptide cycle [36]. However, a FAAL enzyme was also annotated within the putative gene cluster (HasG); thus, the process may be analogous to the one we have reconstructed in our study. A structural motif (3-amino-2-hydroxy-4-methyl fatty acid) identical to puwainaphycin was reported from minutissamides, in which even the length of the FA varied from C14–C18 and furthermore, chlorination and hydroxylation of the FA chain was present [17]. In many cyanobacterial cyclic lipopeptides, the 2-hydroxy group and 4-methyl groups are missing compared with puwainaphycins, and the β-amino FA chain bears one or more hydroxyls, for example, as in pahyokolides [18] and largamide H [21]. Alternatively, some variants of cyanobacterial lipopeptides occur where a sole β-amino group is present in the FA, as was described for laxaphycins [15]. It may be expected that this diversity is generated by different combinations of the FAAL/PKS/oxygenase/aminotransferase enzymatic domains at the beginning of the synthetic pathway. Taking into consideration the multispecificity of the FAAL domain and possible post-synthetic modifications, it is obvious that a large number of lipopeptides with identical amino acid cycles that differ only in FA chains may be generated. Moreover, the substituents on the α and γ carbons of the FA may vary. Thus, we propose designating the lipopeptide by a prefix that denotes the length of the FA and the substituents on the α and β carbon in alphabetical order. All substituents starting from the γ-carbon should be mentioned separately.

Given the similar (although highly modified) mechanism of biosynthesis of puwainaphycins and iturin family antibiotics, it appears that a combination of FAAL and PKS enzymes containing an aminotransferase may be characteristic of bacterial β-amino-lipopeptide synthetases in general. Interestingly, a very similar machinery has recently been proposed to take part in cyanobacterial olefin synthesis [54]. A hybrid enzyme comprising FAAL, ACP, and a PKS was suggested to be employed in one of the two common mechanisms of producing hydrocarbons in cyanobacteria. It can be easily imagined that β-amino-lipopeptide synthesis may have shared a common evolutionary origin with olefin synthesis, differing by the addition of an aminotransferase (and other) domains to the extant enzyme complex. Supporting this hypothesis, the FAAL domain of an olefin synthetase from *Moorea bouillonii* [54] was among the best-scoring blastp hits for the PuwC FAAL enzyme (59% protein sequence identity).

Our results provide the first attempt to describe the biosynthesis of β-amino lipopeptides in cyanobacteria. The only other known type of cyanobacterial lipopeptide operons encodes a different class of lipopeptides, hassallidins. However, the obvious similarity to known lipopeptide biosynthesis machineries in bacteria enables us to discuss the enzymatic steps putatively universal in this kind of compounds and their numerous variants. Once additional data are collected on other similar biosynthetic clusters, molecular markers specific for FAAL and other characteristic enzymatic domains may serve as environmental-monitoring tools for toxic lipopeptide producers or for identification of bioactive lipopeptide producers in antimicrobial research.

## Supporting Information

Figure S1 **Mass spectrum displaying the fragmentation pattern of different puwainaphycin analogs.** (A) 4-methyl-Ahdoa-Puw-F; (B) 4-methyl-Ahtea-Puw-F; (C) 4-methyl-Ahdoa-Puw-G; (D) 4-methyl-Ahtea-Puw-G.
(PDF)

Figure S2 **NMR spectra of 4-methyl-Ahdoa-Puw-F in DMSO.** (A) ^1^H NMR spectrum; (B) 2D ^1^H^13^C HSQC spectrum; (C) 2D ^1^H^15^N HSQC spectrum.
(PDF)

Table S1 Gradient used for pre-purification of the puwainaphycin analogs on preparative C18-column.
(PDF)

Table S2 Gradient used for second purification step of the puwainaphycin analogs on semi-preparative Phenyl-column.
(PDF)

Table S3 Deduced functions of the open reading frames in the *puw* gene cluster.
(PDF)

Table S4 Product ions obtained by high resolution mass spectrometry (MS/MS) experiments performed with different puwainaphycin analogs. Diagnostic fragments are highlighted. (A) 4-methyl-Ahtea-Puw-F; (B) 4-mehtyl-Ahdoa-Puw-F; (C) chloro-4-methyl-Ahtea-Puw-F; (D) chloro-4-methyl-Ahdoa-Puw-F; (E) hydroxy-4-methyl-Ahtea-Puw-F; (F) hydroxy-4-methyl-Ahdoa-Puw-F.
(PDF)

Table S5 NMR assignment of different puwainaphycin variants. The values marked as grey were not proved because of the signal overlap in the mixture of particular F/G variants of puwainaphycin analogs.
(PDF)
