# Supplementary material for: A Hybrid Non-Ribosomal Peptide/Polyketide Synthetase Containing Fatty-Acyl Ligase (FAAL) Synthesizes the β-Amino Fatty Acid Lipopeptides Puwainaphycins in the Cyanobacterium Cylindrospermum alatosporum
Source: PLoS One. 2014 Nov 4;9(11):e111904. doi: 10.1371/journal.pone.0111904 (PMC4219810; doi:10.1371/journal.pone.0111904)

**Figure S1. Mass spectrum displaying the fragmentation pattern of different puwainaphycin analogs.** (A) 4-methyl-Ahdoa-puw-F; (B) 4-methyl-Ahtea-puw-F; (C) 4-methyl-Ahdoa-puw-G; (D) 4-methyl-Ahtea-puw-G.

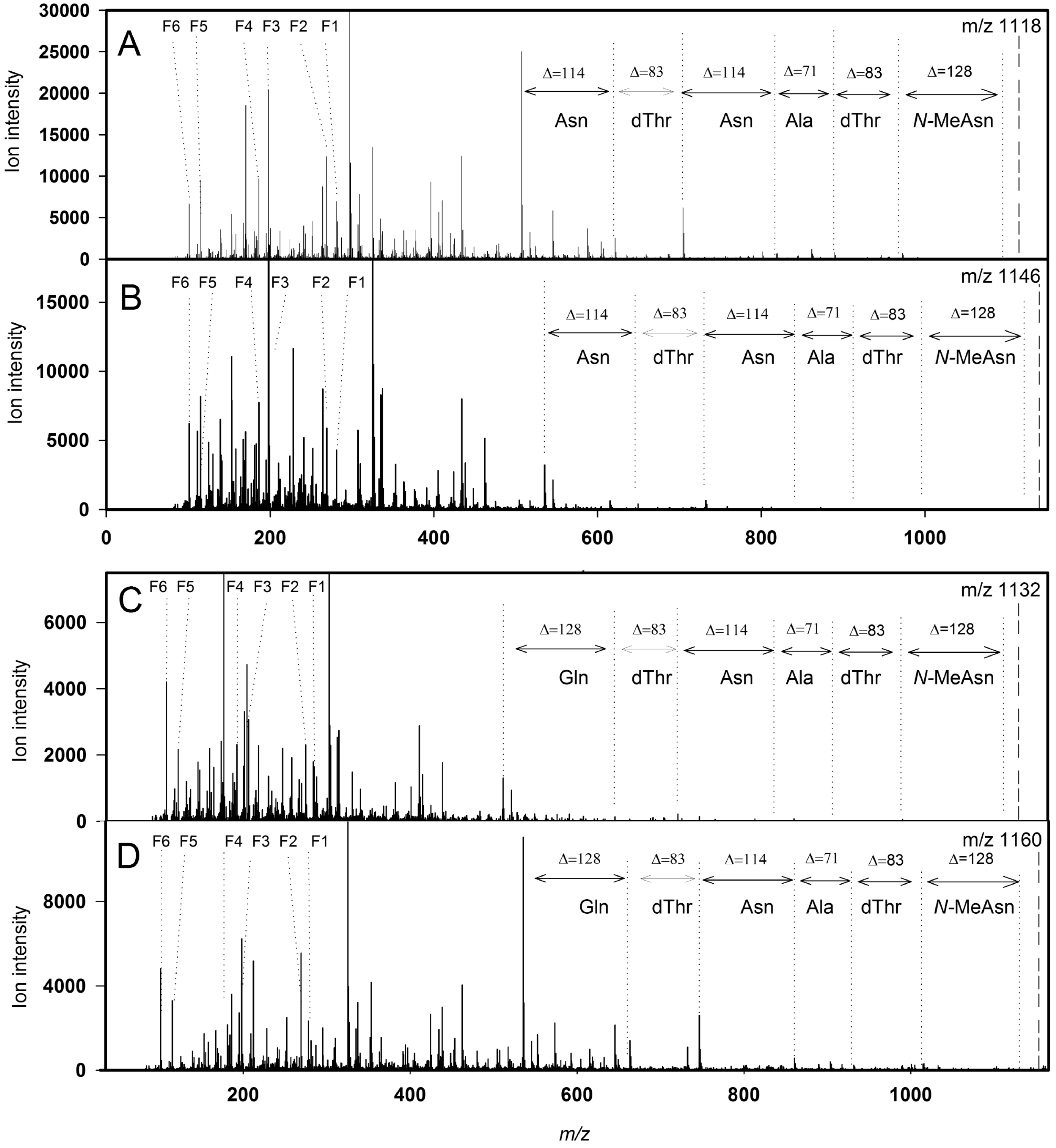

Supplement: Figure S1 — Mass spectrum displaying the fragmentation pattern of different puwainaphycin analogs. (A) 4-methyl-Ahdoa-Puw-F; (B) 4-methyl-Ahtea-Puw-F; (C) 4-methyl-Ahdoa-Puw-G; (D) 4-methyl-Ahtea-Puw-G. (PDF) [file pone.0111904.s001.pdf]
