# Supplementary material for: A Hybrid Non-Ribosomal Peptide/Polyketide Synthetase Containing Fatty-Acyl Ligase (FAAL) Synthesizes the β-Amino Fatty Acid Lipopeptides Puwainaphycins in the Cyanobacterium Cylindrospermum alatosporum
Source: PLoS One. 2014 Nov 4;9(11):e111904. doi: 10.1371/journal.pone.0111904 (PMC4219810; doi:10.1371/journal.pone.0111904)

Figure S2. NMR spectra of 4-methyl-Ahdoa-puw-F in DMSO. (A) ^{1}H NMR spectrum; (B) 2D $^{1}H^{13}C$ HSQC spectrum; (C) 2D $^{1}H^{15}N$ HSQC spectrum.

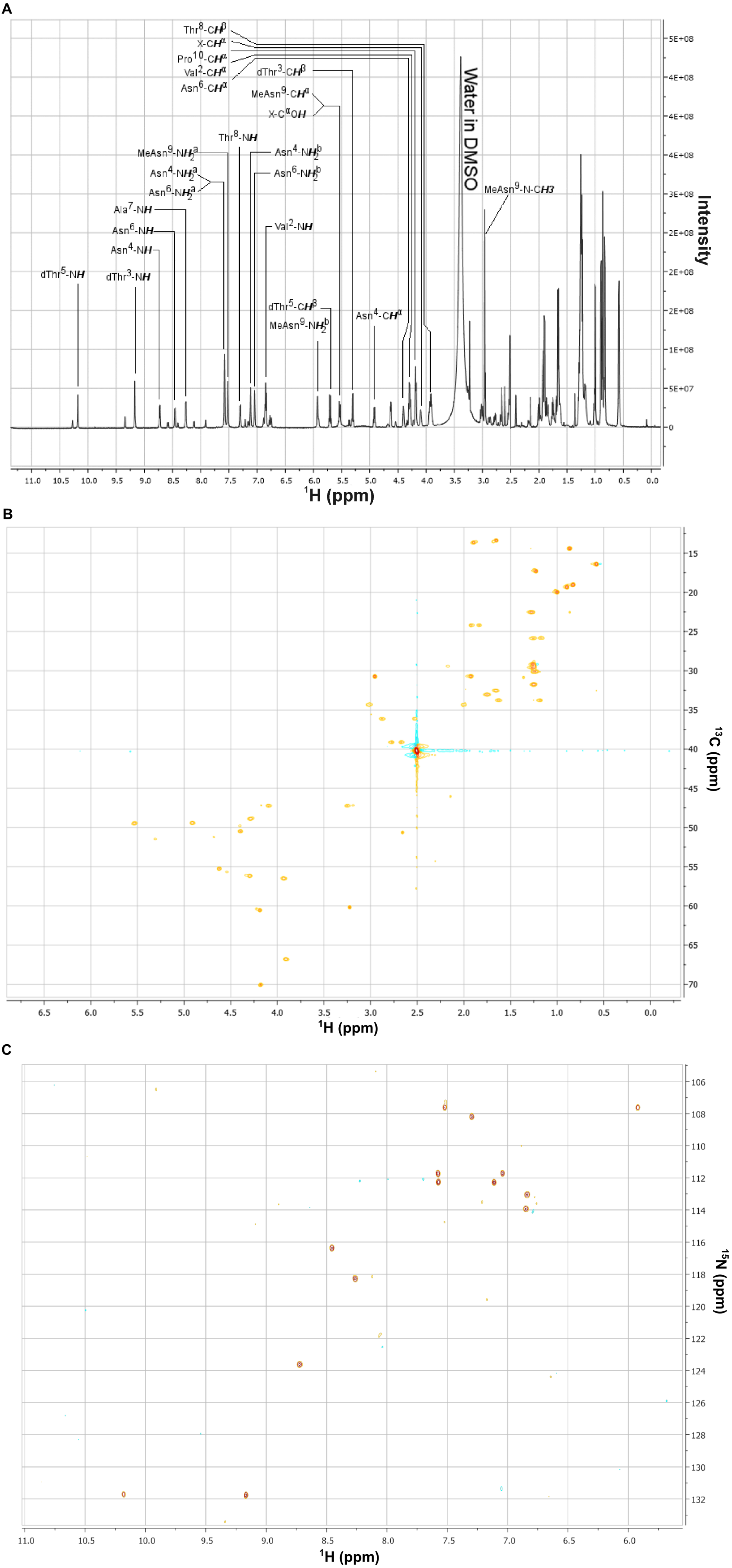

Supplement: Figure S2 — NMR spectra of 4-methyl-Ahdoa-Puw-F in DMSO. (A) 1H NMR spectrum; (B) 2D 1H13C HSQC spectrum; (C) 2D 1H15N HSQC spectrum. (PDF) [file pone.0111904.s002.pdf]
