# Supplementary material for: A Hybrid Non-Ribosomal Peptide/Polyketide Synthetase Containing Fatty-Acyl Ligase (FAAL) Synthesizes the β-Amino Fatty Acid Lipopeptides Puwainaphycins in the Cyanobacterium Cylindrospermum alatosporum
Source: PLoS One. 2014 Nov 4;9(11):e111904. doi: 10.1371/journal.pone.0111904 (PMC4219810; doi:10.1371/journal.pone.0111904)
Supplement: Table S1 — Gradient used for pre-purification of the puwainaphycin analogs on preparative C18-column. (PDF) [file pone.0111904.s003.pdf]

Table S1. Gradient used for pre-purification of the puwainaphycin analogs on preparative C18-column.

Time	H_2O	MeOH
0 min	70%	30%
6 min	70%	30%
15 min	30%	70%
43 min	17%	83%
45 min	0%	100%
52 min	0%	100%
56 min	70%	30%
60 min	70%	30%
