# Supplementary material for: A Hybrid Non-Ribosomal Peptide/Polyketide Synthetase Containing Fatty-Acyl Ligase (FAAL) Synthesizes the β-Amino Fatty Acid Lipopeptides Puwainaphycins in the Cyanobacterium Cylindrospermum alatosporum
Source: PLoS One. 2014 Nov 4;9(11):e111904. doi: 10.1371/journal.pone.0111904 (PMC4219810; doi:10.1371/journal.pone.0111904)
Supplement: Table S2 — Gradient used for second purification step of the puwainaphycin analogs on semi-preparative Phenyl-column. (PDF) [file pone.0111904.s004.pdf]

Table S2. Gradient used for the second purificatin step of the puwainaphycin analogs on semi-preparative Phenyl-column.

Time	H_2O	MeOH
0 min	70%	30%
2 min	70%	30%
6 min	28	62%
26 min	20%	80%
30 min	0%	100%
35 min	0%	100%
40 min	70%	30%
