# Supplementary material for: A Hybrid Non-Ribosomal Peptide/Polyketide Synthetase Containing Fatty-Acyl Ligase (FAAL) Synthesizes the β-Amino Fatty Acid Lipopeptides Puwainaphycins in the Cyanobacterium Cylindrospermum alatosporum
Source: PLoS One. 2014 Nov 4;9(11):e111904. doi: 10.1371/journal.pone.0111904 (PMC4219810; doi:10.1371/journal.pone.0111904)
Supplement: Table S3 — Deduced functions of the open reading frames in the puw gene cluster. (PDF) [file pone.0111904.s005.pdf]

Table S3. Deduced functions of the open reading frames in the *puw* gene cluster.

Protein	Amino Acids	Predicted Function	Similarity (Protein, Origin)	Pairwise Identity	Accession No.	Reference
ORF1	659	ABC transporter	ABC transporter , *Nostoc punctiforme* PCC 73102	53%	ACC81026	[1]
ORF2	1116	patatin-like phospholipase	patatin-like protein, *Nostoc* sp. 152	43%	AGZ05269	[2]
PuwA	2870	NRPS	TubC, *Microcystis aeruginosa* TAIHU98	50%	ELP56284	[3]
PuwB	2534	PKS	JamJ, Lyngbya majuscula JHB	50%	AAS98781	[4]
PuwC	597	fatty acyl-AMP ligase	Fatty acyl ACP ligase, *Moorea bouillonii* PNG5-198	59%	AHH34187	[5]
PuwD	101	acyl carrier protein	acyl carrier protein, *Pleurocapsa* sp. PCC 7327	59%	YP_007079289	Gugger et al. (GenBank data)
PuwE	3077	hybrid PKS/NRPS + aminotransferase, monooxidase	MicA, *Planktothrix prolifica* NIVA-CYA 98	53%	CAQ48259	[6]
PuwF	2370	NRPS	A-domain containing protein, *Nostoc punctiforme* PCC 73102	51%	YP_001866470	Copeland et al. (GenBank data)
PuwG	3492	NRPS	peptide synthetase, *Myxococcus xanthus*	50%	WP_020478358	[7]
PuwH	1102	NRPS	A-domain containing protein, *Nostoc punctiforme* PCC 73102	61%	YP_001866471	Copeland et al. (GenBank data)

1. Ekman M, Picossi S, Campbell EL, Meeks JC, Flores E (2013) A *Nostoc punctiforme* Sugar Transporter Necessary to Establish a Cyanobacterium-Plant Symbiosis. Plant Physiology 161: 1984-1992.

2. Fewer DP, Wahlsten M, Osterholm J, Jokela J, Rouhiainen L, et al. (2013) The Genetic Basis for O-Acetylation of the Microcystin Toxin in Cyanobacteria. Chemistry & Biology 20: 861-869.

3. Yang C, Zhang W, Ren M, Song L, Li T et al. (2013) Whole-Genome Sequence of *Microcystis aeruginosa* TAIHU98, a Nontoxic Bloom-Forming Strain Isolated from Taihu Lake, China. Genome Announcments 1: e00333-13.

4. Edwards DJ, Marquez BL, Nogle LM, McPhail K, Goeger DE et al. (2004) Structure and biosynthesis of the jamaicamides, new mixed polyketide-peptide neurotoxins from the marine cyanobacterium *Lyngbya majuscula*. Chemistry & Biology 11: 817-833.

5. Coates RC, Podell S, Korobeynikov A, Lapidus A, Pevzner P, et al. (2014) Characterization of Cyanobacterial Hydrocarbon Composition and Distribution of Biosynthetic Pathways. Plos One 9.

6. Rounge TB, Rohrlack T, Nederbragt AJ, Kristensen T, Jakobsen KS (2009) A genome-wide analysis of nonribosomal peptide synthetase gene clusters and their peptides in a *Planktothrix rubescens* strain. Bmc Genomics 10.

7. Müller S, Willett JW, Bahr SM, Scott JC, Wilson JM et al. (2013) Draft Genome of a Type 4 Pilus Defective *Myxococcus xanthus* Strain, DZF1 Genome Announcments 1: e00392-13.
