# Supplementary material for: A Hybrid Non-Ribosomal Peptide/Polyketide Synthetase Containing Fatty-Acyl Ligase (FAAL) Synthesizes the β-Amino Fatty Acid Lipopeptides Puwainaphycins in the Cyanobacterium Cylindrospermum alatosporum
Source: PLoS One. 2014 Nov 4;9(11):e111904. doi: 10.1371/journal.pone.0111904 (PMC4219810; doi:10.1371/journal.pone.0111904)
Supplement: Table S4 — Product ions obtained by high resolution mass spectrometry (MS/MS) experiments performed with different puwainaphycin analogs. Diagnostic fragments are highlighted. (A) 4-methyl-Ahtea-Puw-F; (B) 4-mehtyl-Ahdoa-Puw-F; (C) chloro-4-methyl-Ahtea-Puw-F; (D) chloro-4-methyl-Ahdoa-Puw-F; (E) hydroxy-4-methyl-Ahtea-Puw-F; (F) hydroxy-4-methyl-Ahdoa-Puw-F. (PDF) [file pone.0111904.s006.pdf]

Table S4. Product ions obtained by high resolution mass spectrometry (MS/MS) experiments performed with different puwainaphycin analogs. Diagnostic fragments are highlighted. (A) 4-methyl-Ahtea-Puw-F; (B) 4-mehtyl-Ahdoa-Puw-F; (C) chloro-4-methyl-Ahtea-Puw-F; (D) chloro-4-methyl-Ahdoa-Puw-F; (E) hydroxy-4-methyl-Ahtea-Puw-F; (F) hydroxy-4-methyl-Ahdoa-Puw-F.

A) 4-methyl-Ahtea-Puw-F

m/z	Intensity	Formula	Error/mSigma	Interpretation
**1146.6517**		$C_{53}H_{87}N_{13}O_{15}+H^+$	0.0/4.9	cyclic P-*4-methyl-Ahtea*-V-dT-N-dT-N-A-T-NMB
1128.6372	156	$C_{53}H_{86}N_{13}O_{14}+H^+$	3.5/289	P-*4-methyl-Ahtea*-V-dT-N-dT-N-A-dT-NMB
1000.5809	854	$C_{48}H_{77}N_{11}O_{12}+H^+$	1.7/68.2	P-*4-methyl-Ahtea*-V-dT-N-dT-N-A-dT
917.5455	542	$C_{44}H_{72}N_{10}O_{11}+H^+$	0.0/98.2	P-*4-methyl-Ahtea*-V-dT-N-dT-N-A
846.5102	1044	$C_{41}H_{67}N_{9}O_{10}+H^+$	-2.1/30.6	P-*4-methyl-Ahtea*-V-dT-N-dT-N
732.4655	7302	$C_{37}H_{61}N_{7}O_{8}+H^+$	-0.1/15.5	P-*4-methyl-Ahtea*-V-dT-N-dT
649.4275	3748	$C_{33}H_{56}N_{6}O_{7}+H^+$	1.2/52.1	P-*4-methyl-Ahtea*-V-dT-N
535.3846	23412	$C_{29}H_{50}N_{4}O_{5}+H^+$	1.6/19.4	P-*4-methyl-Ahtea*-V-dT
424.354	7424	$C_{24}H_{45}N_{3}O_{3}+H^+$	-1.6/17.1	P-*4-methyl-Ahtea*-V (fragment)
353.2816	9994	$C_{20}H_{36}N_{2}O_{3}+H^+$	-3.9/19.8	P-*4-methyl-Ahtea*-CH(OH)-C(O)
325.2858	55068	$C_{19}H_{36}N_{2}O_{2}+H^+$	-2.5/52.2	P-*4-methyl-Ahtea*-CH(OH)
281.1247	8644	$C_{12}H_{16}N_{4}O_{4}+H^+$	-2.5/15.4	Fragment1
269.1247	10940	$C_{11}H_{16}N_{4}O_{4}+H^+$	-1.0/16.1	Fragment2
198.0878	20258	$C_{8}H_{11}N_{3}O_{3}+H^+$	-2.4/5.0	Fragment3
186.0879	8300	$C_{7}H_{11}N_{3}O_{3}+H^+$	-3.3/24.6	Fragment4
115.0868	9700	$C_{5}H_{10}N_{2}O+H^+$	-1.6/4.2	Fragment5
101.0709	6844	$C_{4}H_{8}N_{2}O+H^+$	-0.4/8.4	Fragment6

B) 4-mehtyl-Ahdoa-Puw-F

m/z	Intensity	Formula	Error/mSigma	Interpretation
**1118.6248**		$C_{51}H_{83}N_{13}O_{15}+H^+$	-3.9/10.2	cyclic P-*4-methyl-Ahdoa*-V-dT-N-dT-N-A-T-NMB
1101.6168	86	$C_{51}H_{82}N_{13}O_{14}+H^+$	0.8/67.2	P-*4-methyl-Ahdoa*-V-dT-N-dT-N-A-dT-NMB
972.5500	378	$C_{46}H_{73}N_{11}O_{12}+H^+$	1.3/24.5	P-*4-methyl-Ahdoa*-V-dT-N-dT-N-A-dT
889.5160	682	$C_{42}H_{68}N_{10}O_{11}+H^+$	-2.0/47.9	P-*4-methyl-Ahdoa*-V-dT-N-dT-N-A
818.4797	1220	$C_{39}H_{63}N_{9}O_{10}+H^+$	-3.2/34.4	P-*4-methyl-Ahdoa*-V-dT-N-dT-N
704.4344	6810	$C_{35}H_{57}N_{7}O_{8}+H^+$	-0.4/24.6	P-*4-methyl-Ahdoa*-V-dT-N-dT
621.3990	1826	$C_{31}H_{52}N_{6}O_{7}+H^+$	-3.1/17.7	P-*4-methyl-Ahdoa*-V-dT-N
507.3555	23966	$C_{27}H_{46}N_{4}O_{5}+H^+$	-2.8/12.3	P-*4-methyl-Ahdoa*-V-dT
396.3225	9556	$C_{22}H_{41}N_{3}O_{3}+H^+$	-1.0/26.5	P-*4-methyl-Ahdoa*-V (fragment)
325.2506	12728	$C_{18}H_{32}N_{2}O_{3}+H^+$	-6.2/21.7	P-*4-methyl-Ahdoa*-CH(OH)-C(O)
297.2548	60834	$C_{17}H_{32}N_{2}O_{2}+H^+$	-3.7/70.2	P-*4-methyl-Ahdoa*-CH(OH)
281.1255	7322	$C_{12}H_{16}N_{4}O_{4}+H^+$	-3.6/50.4	Fragment1
269.1256	10726	$C_{11}H_{16}N_{4}O_{4}+H^+$	-4.2/11.9	Fragment2
198.0880	21394	$C_{8}H_{11}N_{3}O_{3}+H^+$	-3.5/8.5	Fragment3
186.0880	7870	$C_{7}H_{11}N_{3}O_{3}+H^+$	-3.7/15.7	Fragment4
115.0872	10834	$C_{5}H_{10}N_{2}O+H^+$	-5.7/2.5	Fragment5
101.0713	6330	$C_{4}H_{8}N_{2}O+H^+$	-3.2/12.7	Fragment6

C) chloro-4-methyl-Ahtea-Puw-F

m/z	Intensity	Formula	Error/mSigma	Interpretation
**1180.6136**		$C_{53}H_{86}ClN_{13}O_{15}+H^+$	-0.7/28.7	cyclic P-*chloro-4-methyl-Ahtea*-V-dT-N-dT-N-A-T-NMB
1163.5931	388	$C_{53}H_{85}ClN_{13}O_{14}+H^+$	0.4/181.5	P-*chloro-4-methyl-Ahtea*-V-dT-N-dT-N-A-dT-NMB
1034.5425	912	$C_{48}H_{76}ClN_{11}O_{12}+H^+$	1.1/115.5	P-*chloro-4-methyl-Ahtea*-V-dT-N-dT-N-A-dT
951.5154	588	$C_{44}H_{71}ClN_{10}O_{11}+H^+$	-1.8/103	P-*chloro-4-methyl-Ahtea*-V-dT-N-dT-N-A
880.4685	470	$C_{41}H_{66}ClN_{9}O_{10}+H^+$	0.4/129	P-*chloro-4-methyl-Ahtea*-V-dT-N-dT-N
766.4269	2746	$C_{37}H_{60}ClN_{7}O_{8}+H^+$	-0.6/68.7	P-*chloro-4-methyl-Ahtea*-V-dT-N-dT
683.3903	1361	$C_{33}H_{55}ClN_{6}O_{7}+H^+$	-1.4/47.4	P-*chloro-4-methyl-Ahtea*-V-dT-N
569.3458	5800	$C_{29}H_{49}ClN_{4}O_{5}+H^+$	1.2/48.7	P-*chloro-4-methyl-Ahtea*-V-dT
533.3718	1926	$C_{29}H_{48}N_{4}O_{5}+H^+$	-3.8/71.0	P-*4-methyl-Ahtea*(unsaturated)-V-dT
458.3129	1388	$C_{24}H_{44}ClN_{3}O_{3}+H^+$	3.2/116.3	P-*chloro-4-methyl-Ahtea*-V (fragment)
387.2403	992	$C_{20}H_{36}ClN_{2}O_{3}+H^+$	-1.5/31.5	P-*chloro-4-methyl-Ahtea*-CH(OH)-C(O)
359.2454	2186	$C_{19}H_{35}ClN_{2}O_{2}+H^+$	1.6/47.5	P-*chloro-4-methyl-Ahtea*-CH(OH)
323.2696	2136	$C_{19}H_{34}N_{2}O_{2}+H^+$	-1.1/132.7	P-*4-methyl-Ahtea*(unsaturated)-CH(OH)
281.1252	1328	$C_{12}H_{16}N_{4}O_{4}+H^+$	-2.6/135	Fragment1
269.1248	2670	$C_{11}H_{16}N_{4}O_{4}+H^+$	-1.4/36.8	Fragment2
198.0875	2856	$C_{8}H_{11}N_{3}O_{3}+H^+$	-1.0/9.5	Fragment3
186.0872	1228	$C_{7}H_{11}N_{3}O_{3}+H^+$	0.6/52.8	Fragment4
115.0863	1272	$C_{5}H_{10}N_{2}O+H^+$	2.6/n.a	Fragment5
101.0700	1418	$C_{4}H_{8}N_{2}O+H^+$	9.3/3.6	Fragment6

D) hydroxy-4-methyl-Ahtea-Puw-F

m/z	Intensity	Formula	Error/mSigma	Interpretation
**1152.5822**		$C_{51}H_{82}ClN_{13}O_{15}+H^+$	-0.6/56.4	cyclic P-*chloro-4-methyl-Ahdoa*-V-dT-N-dT-N-A-T-NMB
1135.5714	300	$C_{51}H_{80}ClN_{13}O_{14}+H^+$	6.5/n.a	P-*chloro-4-methyl-Ahdoa*-V-dT-N-dT-N-A-dT-NMB
1006.5135	236	$C_{41}H_{72}ClN_{13}O_{14}+H^+$	-5.2/n.a	P-*chloro-4-methyl-Ahdoa*-V-dT-N-dT-N-A-dT
923.466	396	$C_{42}H_{67}ClN_{10}O_{11}+H^+$	10.0/215	P-*chloro-4-methyl-Ahdoa*-V-dT-N-dT-N-A
852.442	960	$C_{39}H_{62}ClN_{9}O_{10}+H^+$	-4.6/226.8	P-*chloro-4-methyl-Ahdoa*-V-dT-N-dT-N
738.3985	4420	$C_{35}H_{56}ClN_{7}O_{8}+H^+$	-4.5/191.1	P-*chloro-4-methyl-Ahdoa*-V-dT-N-dT
655.3588	1746	$C_{31}H_{51}ClN_{6}O_{7}+H^+$	-1.1/49.3	P-*chloro-4-methyl-Ahdoa*-V-dT-N
541.3145	14134	$C_{27}H_{45}ClN_{4}O_{5}+H^+$	1.1/46.8	P-*chloro-4-methyl-Ahdoa*-V-dT
505.3412	696	$C_{27}H_{44}N_{4}O_{5}+H^+$	-5.5/234	P-*4-methyl-Ahdoa*(unsaturated)-V-dT
430.2834	4752	$C_{22}H_{40}ClN_{3}O_{3}+H^+$	-0.7/60.6	P-*chloro-4-methyl-Ahdoa*-V (fragment)
359.2092	4722	$C_{18}H_{31}ClN_{2}O_{3}+H^+$	1.2/39.7	P-*chloro-4-methyl-Ahdoa*-CH(OH)-C(O)
331.2142	17988	$C_{17}H_{31}ClN_{2}O_{2}+H^+$	1.4/63.8	P-*chloro-4-methyl-Ahdoa*-CH(OH)
295.2355	1452	$C_{17}H_{30}N_{2}O_{2}+H^+$	8.5/33.5	P-*4-methyl-Ahdoa*(unsaturated) -CH(OH)
281.1237	5226	$C_{12}H_{16}N_{4}O_{4}+H^+$	2.8/49.9	Fragment1
269.1234	10494	$C_{11}H_{16}N_{4}O_{4}+H^+$	4.0/n.a.	Fragment2
198.0867	14250	$C_{8}H_{11}N_{3}O_{3}+H^+$	3.4/6.6	Fragment3
186.0865	6894	$C_{7}H_{11}N_{3}O_{3}+H^+$	4.1/6.2	Fragment4
115.0862	7700	$C_{5}H_{10}N_{2}O+H^+$	3.0/2.7	Fragment5
101.0713	6788	$C_{4}H_{8}N_{2}O+H^+$	-3.4/21.9	Fragment6

E) chloro-4-methyl-Ahdoa-Puw-F

m/z	Intensity	Formula	Error/mSigma	Interpretation
**1162.6451**		$C_{53}H_{87}N_{13}O_{16}+H^{+}$	1.3/55.5	cyclic P-*hydroxy-4-methyl-Ahtea*-V-dT-N-dT-N-A-T-NMB
1144.6421	350	$C_{53}H_{85}N_{13}O_{15}+H^{+}$	-5.3/316	P-*hydroxy-4-methyl-Ahtea*-V-dT-N-dT-N-A-dT-NMB
1016.5799	260	$C_{48}H_{77}N_{11}O_{13}+H^{+}$	-2.3/173	P-*hydroxy-4-methyl-Ahtea*-V-dT-N-dT-N-A-dT
933.5439	136	$C_{44}H_{72}N_{10}O_{12}+H^{+}$	-3.7/n.a.	P-*hydroxy-4-methyl-Ahtea*-V-dT-N-dT-N-A
862.5005	174	$C_{41}H_{67}N_{9}O_{11}+H^{+}$	3.2/n.a.	P-*hydroxy-4-methyl-Ahtea*-V-dT-N-dT-N
748.4589	742	$C_{37}H_{61}N_{7}O_{9}+H^{+}$	2.0/59.3	P-*hydroxy-4-methyl-Ahtea*-V-dT-N-dT
665.4200	428	$C_{33}H_{56}N_{6}O_{8}+H^{+}$	4.9/218	P-*hydroxy-4-methyl-Ahtea*-V-dT-N
551.3821	570	$C_{29}H_{50}N_{4}O_{6}+H^{+}$	-3.3/39.4	P-*hydroxy-4-methyl-Ahtea*-V-dT
533.3696	430	$C_{29}H_{48}N_{4}O_{5}+H^{+}$	0.2/n.a.	P-*4-methyl-Ahtea*(unsaturated)-V-dT
440.3469	256	$C_{24}H_{45}N_{3}O_{4}+H^{+}$	3.2/n.a.	P-*hydroxy-4-methyl-Ahtea*-V (fragment)
369.2752	182	$C_{20}H_{36}N_{2}O_{4}+H^{+}$	-1.0/97.9	P-*hydroxy-4-methyl-Ahtea*-CH(OH)-C(O)
341.2782	220	$C_{19}H_{36}N_{2}O_{3}+H^{+}$	4.8/n.a.	P-*hydroxy-4-methyl-Ahtea*-CH(OH)
323.2690	134	$C_{19}H_{34}N_{2}O_{2}+H^{+}$	0.9/n.a.	P-*4-methyl-Ahtea*(unsaturated)-CH(OH)
281.1241	246	$C_{12}H_{16}N_{4}O_{4}+H^{+}$	1.3/n.a.	Fragment1
269.1252	482	$C_{11}H_{16}N_{4}O_{4}+H^{+}$	-2.8/21.4	Fragment2
198.0880	246	$C_{8}H_{11}N_{3}O_{3}+H^{+}$	-3.4/n.a.	Fragment3
186.0871	204	$C_{7}H_{11}N_{3}O_{3}+H^{+}$	0.9/n.a.	Fragment4
115.0853	90	$C_{5}H_{10}N_{2}O+H^{+}$	11.2/n.a.	Fragment5
101.0700	164	$C_{4}H_{8}N_{2}O+H^{+}$	8.9/n.a.	Fragment6

F) hydroxy-4-methyl-Ahdoa-Puw-F

m/z	Intensity	Formula	Error/mSigma	Interpretation
**1134.6146**		$C_{51}H_{83}N_{13}O_{16}+H^{+}$	0.6/52.1	cyclic P-*hydroxy-4-methyl-Ahdoa*-V-dT-N-dT-N-A-T-NMB
1117.6049	183	$C_{51}H_{82}N_{13}O_{15}+H^{+}$	6.9/96.4	P-*hydroxy-4-methyl-Ahdoa*-V-dT-N-dT-N-A-dT-NMB
988.5561	160	$C_{41}H_{73}N_{13}O_{15}+H^{+}$	-14.1/n.a	P-*hydroxy-4-methyl-Ahdoa*-V-dT-N-dT-N-A-dT
905.501	332	$C_{41}H_{66}N_{11}O_{12}+H^{+}$	-5.0/237.9	P-*hydroxy-4-methyl-Ahdoa*-V-dT-N-dT-N-A
834.476	440	$C_{39}H_{63}N_{9}O_{11}+H^{+}$	-4.9/71.7	P-*hydroxy-4-methyl-Ahdoa*-V-dT-N-dT-N
720.4277	1750	$C_{35}H_{58}N_{7}O_{9}+H^{+}$	1.9/180.7	P-*hydroxy-4-methyl-Ahdoa*-V-dT-N-dT
637.3907	1334	$C_{31}H_{52}N_{6}O_{8}+H^{+}$	2.0/89.2	P-*hydroxy-4-methyl-Ahdoa*-V-dT-N
523.3477	2540	$C_{27}H_{46}N_{4}O_{6}+H^{+}$	2.5/59.8	P-*hydroxy-4-methyl-Ahdoa*-V-dT
505.3375	766	$C_{27}H_{44}N_{4}O_{5}+H^{+}$	-5.7/90.8	P-*4-methyl-Ahdoa*(unsaturated)-V-dT
412.3179	718	$C_{22}H_{41}N_{3}O_{4}+H^{+}$	-2.2/40.3	P-*hydroxy-4-methyl-Ahdoa*-V (fragment)
341.2442	352	$C_{18}H_{32}N_{2}O_{4}+H^{+}$	-2.1/203	P-*hydroxy-4-methyl-Ahdoa*-CH(OH)-C(O)
313.2473	4152	$C_{17}H_{32}N_{2}O_{3}+H^{+}$	3.3/86.7	P-*hydroxy-4-methyl-Ahdoa*-CH(OH)
295.2376	1026	$C_{17}H_{30}N_{2}O_{2}+H^{+}$	1.3/46.5	P-*4-methyl-Ahdoa*(unsaturated) -CH(OH)
281.1261	672	$C_{12}H_{16}N_{4}O_{4}+H^{+}$	-6.0/n.a	Fragment1
269.1245	1522	$C_{11}H_{16}N_{4}O_{4}+H^{+}$	-0.2/16.6	Fragment2
198.0873	1726	$C_{8}H_{11}N_{3}O_{3}+H^{+}$	1.5/8.5	Fragment3
186.0863	1292	$C_{7}H_{11}N_{3}O_{3}+H^{+}$	5.7/n.a	Fragment4
115.0867	992	$C_{5}H_{10}N_{2}O+H^{+}$	-0.9/n.a.	Fragment5
101.0708	574	$C_{4}H_{8}N_{2}O+H^{+}$	1.2/31.7	Fragment6
