# Supplementary material for: A Hybrid Non-Ribosomal Peptide/Polyketide Synthetase Containing Fatty-Acyl Ligase (FAAL) Synthesizes the β-Amino Fatty Acid Lipopeptides Puwainaphycins in the Cyanobacterium Cylindrospermum alatosporum
Source: PLoS One. 2014 Nov 4;9(11):e111904. doi: 10.1371/journal.pone.0111904 (PMC4219810; doi:10.1371/journal.pone.0111904)
Supplement: Table S5 — NMR assignment of different puwainaphycin variants. The values marked as grey were not proved because of the signal overlap in the mixture of particular F/G variants of puwainaphycin analogs. (PDF) [file pone.0111904.s007.pdf]

**Table S5. NMR assignment of different puwainaphycin variants.** The values marked as grey were not proven because of the signal overlap in the mixture of particular F/G variants of puwainaphycin analogs.

| | | *4-methyl-Ahdoa*- Puw-F | | | *4-methyl-Ahdoa* -Puw-G | | | *4-methyl-Ahtea* -Puw-F | | | *4-methyl-Ahtea-Puw* -G | | |
|---|---|---|---|---|---|---|---|---|---|---|---|---|---|
| | | δC [ppm] | δH [ppm] | δN [ppm] | δC [ppm] | δH [ppm] | δN [ppm] | δC [ppm] | δH [ppm] | δN [ppm] | δC [ppm] | δH [ppm] | δN [ppm] |
| X | α | 70.08 | 4.18 | | 70.08 | 4.18 | | 69.70 | 4.18 | | 69.77 | 4.15 | |
| | β | 56.53 | 3.93 | | 56.20 | 4.34 | | 56.18 | 3.93 | | 56.06 | 4.32 | |
| | γ | 32.57 | 1.66 | | 32.57 | 1.66 | | 32.20 | 1.66 | | 32.13 | 1.67 | |
| | δ | 33.82 | 1.63 | | 33.83 | 1.61 | | 33.43 | 1.62 | | 33.43 | 1.62 | |
| | | | 1.18 | | | 1.25 | | | 1.17 | | | 1.18 | |
| | $CH_2^1$ | 31.79 | 1.26 | | 31.79 | 1.26 | | 29.71 | 1.29 | | 29.74 | 1.29 | |
| | $CH_2^2$ | 30.15 | 1.24 | | 30.15 | 1.24 | | 29.27 | 1.25 | | 29.27 | 1.25 | |
| | $CH_2^3$ | 29.63 | 1.27 | | 29.63 | 1.27 | | 29.14 | 1.15 | | 29.08 | 1.15 | |
| | $CH_2^4$ | 29.20 | 1.26 | | 29.20 | 1.26 | | 29.10 | 1.24 | | 29.06 | 1.25 | |
| | $CH_2^5$ | 25.86 | 1.18, 1.26 | | 25.86 | 1.18 | | 28.77 | 1.26 | | 28.74 | 1.26 | |
| | $CH_2^6$ | 22.57 | 1.28 | | 22.57 | 1.28 | | 25.45 | 1.18 | | 25.45 | 1.25 | |
| | $CH_2^7$ | X | X | | X | X | | 31.35 | 1.23 | | 31.35 | 1.23 | |
| | $CH_2^8$ | X | X | | X | X | | 22.15 | 1.26 | | 22.15 | 1.26 | |
| | n-$CH_3$ | 14.43 | 0.87 | | 14.43 | 0.87 | | 14.01 | 0.86 | | 14.01 | 0.86 | |
| | α-OH | | 5.54 | | | 5.54 | | | 5.47 | | | 5.56 | |
| | β-NH | | 6.84 | 113.09 | | 6.84 | 113.09 | | 6.83 | 114.84 | | 6.77 | 114.84 |
| | γ-CH3 | 16.44 | 0.58 | | 16.44 | 0.61 | | 16.02 | 0.58 | | 16.02 | 0.57 | |
| Val[2] | NH | | 6.85 | 113.97 | | 6.85 | 113.97 | | 6.86 | 115.71 | | 6.77 | 115.71 |
| | α | 56.18 | 4.30 | | 56.10 | 4.34 | | 55.82 | 4.29 | | 55.73 | 4.33 | |
| | β | 33.08 | 1.75 | | 33.08 | 1.75 | | 32.63 | 1.75 | | 32.68 | 1.72 | |
| | $γ^1$ | 19.39 | 0.90 | | 19.39 | 0.90 | | 18.97 | 0.89 | | 18.80 | 0.88 | |
| | $γ^2$ | 19.09 | 0.83 | | 19.09 | 0.83 | | 18.64 | 0.82 | | 18.70 | 0.82 | |
| dThr[3] | NH | | 9.17 | 131.76 | | 9.17 | 131.76 | | 9.15 | 133.50 | | 9.32 | 133.50 |
| | α | | | | | | | 132.59 | | | 132.39 | | |
| | β | | 5.29 | | | 5.29 | | 116.07 | 5.30 | | 117.40 | 5.36 | |
| | γ | 13.44 | 1.65 | | 13.63 | 1.69 | | 13.02 | 1.65 | | 13.19 | 1.68 | |
| Asn[4] | NH | | 8.72 | 123.64 | | | | | 8.71 | 125.31 | | | |
| | α | 49.45 | 4.91 | | | | | 49.03 | 4.92 | | | | |
| | β | 39.18 | 2.78 | | | | | 38.78 | 2.76 | | | | |
| | | | 2.68 | | | | | | 2.67 | | | | |
| | $NH_2$ | | 7.11 | 112.28 | | | | | 7.54 | 113.87 | | | |
| | | | 7.57 | | | | | | 7.07 | | | | |
| Gln[4] | NH | | | | | 8.72 | 123.64 | | | | | 8.56 | |
| | α | | | | 51.25 | 4.68 | | | | | 50.86 | 4.68 | |
| | β | | | | 29.47 | 2.18 | | | | | 29.06 | 2.17 | |
| | γ | | | | 32.42 | 2.38 | | | | | 32.01 | 2.38 | |
| | | | | | | 1.98 | | | | | | 1.98 | |
| | $NH_2$ | | | | | 7.11 | 112.28 | | | | | 6.86 | |
| | | | | | | 7.57 | | | | | | 6.72 | |
| dThr[5] | NH | | 10.18 | 131.75 | | 10.18 | 131.75 | | 10.15 | 133.26 | | 10.24 | 133.26 |
| | α | | | | | | | 129.69 | | | 129.80 | | |
| | β | | 5.70 | | | 5.70 | | 125.26 | 5.71 | | 124.50 | 5.72 | |
| | γ | 13.67 | 1.90 | | 13.67 | 1.90 | | 13.27 | 1.89 | | 13.21 | 1.87 | |
| Asn[6] | NH | | 8.45 | 116.41 | | 8.45 | 116.41 | | 8.38 | 118.17 | | 7.48 | 118.17 |
| | α | 50.52 | 4.40 | | 49.84 | 4.40 | | 50.11 | 4.40 | | 49.43 | 4.40 | |
| | β | 36.18 | 2.88 | | 36.18 | 2.88 | | 35.80 | 2.87 | | 35.23 | 3.01 | |
| | $NH_2$ | | 7.04 | 111.76 | | 7.04 | 111.76 | | 7.54 | 113.41 | | 7.88 | |
| | | | 7.57 | | | 7.57 | | | 7.01 | | | 7.18 | |
| Ala[7] | NH | | 8.26 | 118.31 | | 8.26 | 118.31 | | 8.25 | 120.07 | | 8.11 | 120.07 |
| | α | 48.52 | 4.29 | | 48.52 | 4.29 | | 48.56 | 4.28 | | 48.44 | 4.27 | |
| | β | 17.35 | 1.23 | | 17.35 | 1.23 | | 16.93 | 1.23 | | 16.02 | 1.24 | |
| Thr[8] | NH | | 7.29 | 108.24 | | 7.29 | 108.24 | | 7.27 | 109.85 | | 7.14 | 109.85 |
| | α | 55.27 | 4.63 | | 55.67 | 4.54 | | 54.82 | 4.63 | | 55.25 | 4.54 | |
| | β | 66.81 | 3.91 | | 66.81 | 3.91 | | 66.43 | 3.90 | | 66.50 | 3.85 | |
| | γ | 20.02 | 1.00 | | 20.02 | 1.00 | | 19.62 | 1.00 | | 19.45 | 1.01 | |
| | β-OH | | | | | | | | 5.07 | | | 5.02 | |
| MeAsn[9] | α | 49.48 | 5.54 | | 49.48 | 5.54 | | 49.12 | 5.53 | | 49.16 | 5.55 | |
| | β | 34.40 | 3.02 | | 34.40 | 3.02 | | 33.97 | 3.01 | | 33.76 | 2.99 | |
| | | | 2.01 | | | 2.01 | | | 2.00 | | | 1.98 | |
| | $NH_2$ | | 5.92 | 107.67 | | 5.92 | 107.67 | | 7.49 | 109.24 | | 7.48 | 109.24 |
| | | | 7.52 | | | 7.52 | | | 5.93 | | | 5.91 | |
| | N-$CH_3$ | 30.76 | 2.96 | | 30.76 | 2.96 | | 30.36 | 2.95 | | 30.39 | 2.94 | |
| Pro[10] | α | 60.57 | 4.19 | | 60.41 | 4.23 | | 60.14 | 4.19 | | 59.99 | 4.22 | |
| | β | 30.75 | 1.93 | | 30.75 | 1.93 | | 30.32 | 1.92 | | 30.32 | 1.92 | |
| | γ | 24.24 | 1.84 | | 24.24 | 1.84 | | 23.82 | 1.91 | | 23.46 | 1.86 | |
| | | | 1.92 | | | 1.92 | | | 1.83 | | | 1.72 | |
| | δ | 47.27 | 3.25 | | 47.27 | 3.19 | | 46.85 | 4.09 | | 46.81 | 4.16 | |
| | | | 4.10 | | | 4.17 | | | 3.25 | | | 3.20 | |
